# Unusual Clinical Manifestations of Thyroid Carcinoma

**DOI:** 10.7759/cureus.37474

**Published:** 2023-04-12

**Authors:** Sharmela Brijmohan, Marwa Elsheikh, Chelsey B Hemmings, Natasha Rastogi, Atara Schultz

**Affiliations:** 1 Internal Medicine, Englewood Health and Medical Center, Englewood, USA; 2 Endocrinology, Summit Health, Englewood, USA

**Keywords:** poorly differentiated thyroid carcinoma, clinical manifestation, scalp lesion, thyroid nodule, hyperparathyroidism, fna, thyroid cancer

## Abstract

Thyroid cancer is considered the most common endocrine malignancy, with the most frequent presentation of differentiated thyroid cancer being a neck swelling or an incidental finding of a thyroid nodule on imaging. In this case series, we describe three cases of thyroid cancer with unusual clinical manifestations. The first case describes a patient who underwent parathyroidectomy for primary hyperparathyroidism and was found to have papillary thyroid cancer on a cervical lymph node biopsy. While this may be coincidental, the literature raises the question of whether there may be an association. The second case describes a patient who presents with a suspicious thyroid nodule and was subsequently diagnosed with follicular thyroid cancer on biopsy. This raises the question of performing thyroidectomy early in patients with a suspicious thyroid nodule but a false negative biopsy. The third case describes a patient with a scalp lesion found to have poorly differentiated thyroid carcinoma, a rare presentation of this form of cancer.

## Introduction

As the most common cancer of the endocrine system, thyroid carcinoma represents 3.8% of all new cancer cases in the United States and is the ninth most common cancer in general [1]. Thyroid malignancies are divided into papillary carcinomas (80%), follicular carcinomas (10%), medullary thyroid carcinomas (5-10%), anaplastic carcinomas (1-2%), primary thyroid lymphomas (rare), and primary thyroid sarcomas (rare) [2]. Differentiated thyroid cancer commonly presents as neck swelling (detected by the patient or clinician) or as an incidentally discovered thyroid nodule on neck imaging [3]. In this case series, we describe three cases of thyroid cancer with unusual clinical manifestations.

## Case presentation

Case 1

A 68-year-old man with a medical history of type II diabetes mellitus and hypertension presented with hypercalcemia. He was diagnosed with primary hyperparathyroidism with laboratory results shown in Table 1. Thyroid ultrasound showed small calcified bilateral thyroid nodules with enlarged hypoechoic cervical lymph nodes and a 1.1 cm solid appearing nodule in the lower pole of the right lobe of the thyroid which could represent a parathyroid adenoma. Nuclear medicine (NM) parathyroid imaging with single-photon emission computed tomography (SPECT) showed faint uptake inferiorly and laterally to the right thyroid. He had no history of nephrolithiasis or fractures, but his calcium level was high enough to recommend surgery. He underwent parathyroidectomy of the right superior and inferior parathyroid glands and cervical lymph node biopsy. However, the biopsy result of a right cervical lymph node was found to be positive for papillary thyroid cancer. The patient subsequently underwent a total thyroidectomy and was found to have multifocal papillary carcinoma involving the right and left thyroid lobes and isthmus; no perineurial invasion or extrathyroidal tumor extension was found.

**Table 1:** Laboratory findings of case 1

Lab Test	Results	Reference values
Serum Calcium	11.3 mg/dl	8.6 -10.3 mg/dl
24hr Urine calcium	363 mg/24hr	55-300 mg/24hr
Parathyroid Hormone (PTH)	65 pg/ml	14- 64 pg/ml
Serum Phosphorous	3.5 mg/dl	2.1- 4.3 mg/dl
25 (OH) Vitamin D	34 ng/ml	30 -100 ng/ml

Case 2

**Figure 1:**
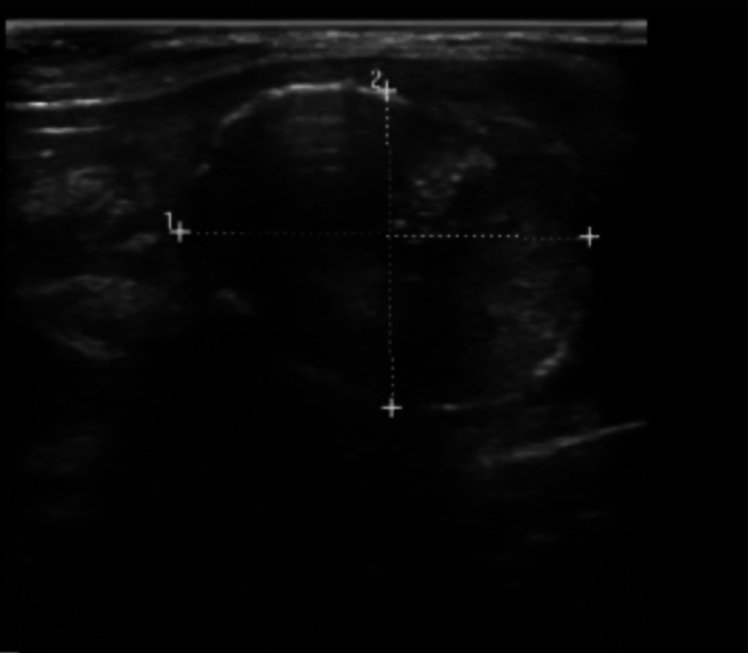
Thyroid nodule on ultrasound

A 73-year-old woman presented with a left thyroid mid pole, heterogeneous and solid nodule with incomplete rim calcification measuring 2.4 x 1.9 x 1.9 cm, and a palpable left cervical level II lymph node measuring 3 x 1.3 x 1 cm (Figure 1). She underwent ultrasound-guided fine-needle aspiration biopsy (FNAB) in 2004 with benign pathology. The repeat ultrasound in 2016 showed that the nodule had increased in size to 3.2 x 2.3 x 1.9 cm. She underwent another biopsy which also yielded benign pathology. The patient was lost to follow-up but re-presented in 2021 and underwent a repeat ultrasound-guided fine-needle aspiration due to an increased nodule size on repeat ultrasound. This biopsy showed a follicular lesion of undetermined significance (Bethesda Diagnostic category III). While the lymph node aspirate did not show cytological evidence of malignancy, a thyroglobulin level in the lymph node aspirate of 13.4 ng/ml, identified covert metastatic thyroid carcinoma. The patient underwent total thyroidectomy with pathology results positive for papillary carcinoma.

Case 3

**Figure 2:**
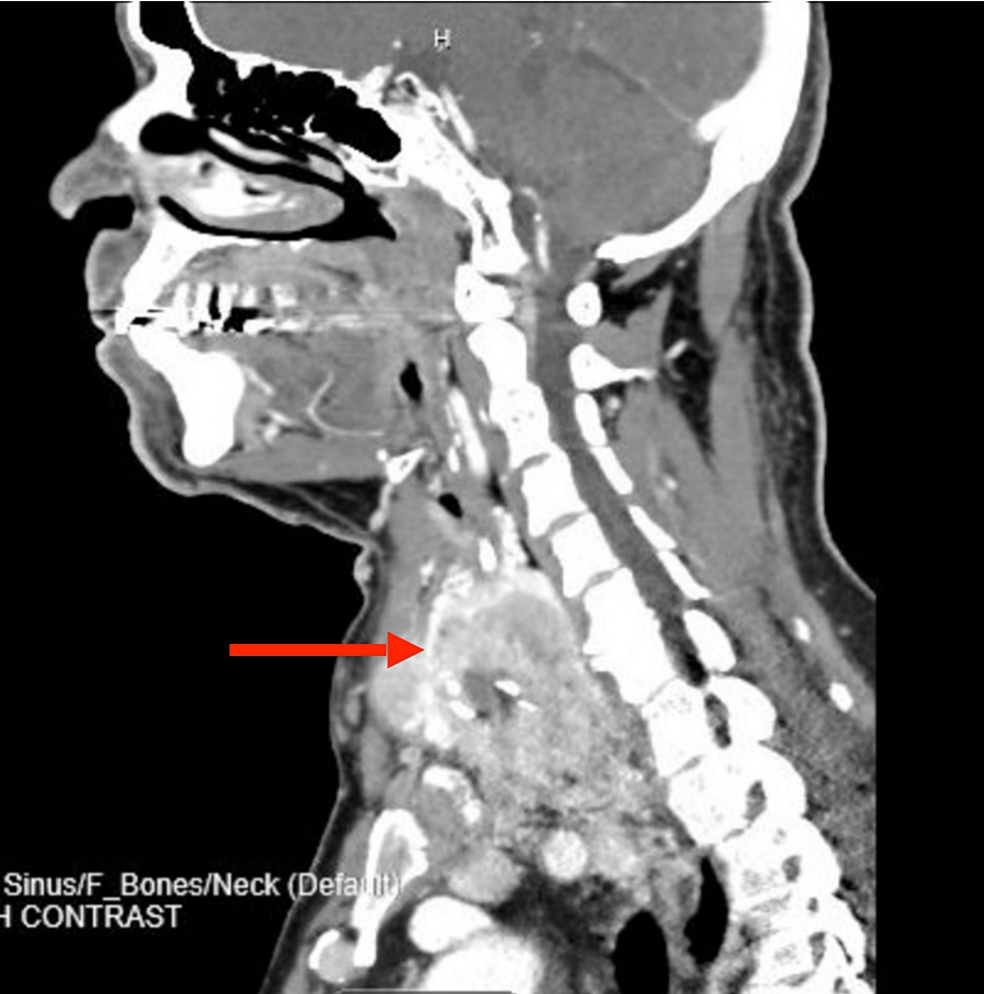
CT neck showing thyroid mass

A 68-year-old man with no significant past medical history presented for investigation of a scalp lesion. Biopsy of the lesion revealed metastatic carcinoma. Immunohistochemical staining was positive for CK7, CAm5.2, TTF-1 and PAX 8, favoring metastatic carcinoma of thyroid origin. CT neck/abdomen/pelvis and thyroid ultrasound showed an expansile infiltrative mass within the right lobe of the thyroid extending to the left lobe and displacing the trachea and esophagus to the left as well as enlarged right hilar and infrahilar lymph nodes. There was no evidence of metastatic disease in the abdomen or pelvis (Figure 2). The patient subsequently underwent total thyroidectomy and neck dissection. Pathology revealed poorly differentiated thyroid carcinoma with predominantly solid-insular, trabecular growth patterns and a rare focus of classic papillary structures. The tumor extended into benign thyroid parenchyma and adjacent peri-thyroid soft tissue into peripheral resection margins. Lymphovascular invasion was present with seven out of nine cervical lymph nodes positive for metastatic carcinoma (T3N1bM1). Levothyroxine was initiated postoperatively at 100 mcg. A full-body nuclear scan one month later showed uptake in the superior mediastinum, liver and spine. The patient received Iodine-131 and further treatment and testing are pending.

## Discussion

In case 1, the patient presents with primary hyperparathyroidism (PHPT) and small unsuspicious thyroid nodules that would usually not be evaluated further. However, his cervical lymph nodes and thyroid gland revealed papillary thyroid cancer (PTC) in postoperative pathology. Indolent neoplasms, like PTC, are often asymptomatic and discovered incidentally. Lombardi et al. reported the proportion of incidental PTC to be 42% [4]. In general, incidental tumor foci are relatively smaller in size (<6 mm) than nonincidental tumor foci [5]. Despite this, further research is needed to determine the clinical significance of concomitant thyroid cancer and hyperparathyroidism. PHPT is not considered a risk factor for thyroid cancer by the ATA guidelines. However, Beebeejaun et al. suggest a possible hypothesis for this risk based on shared embryological origin and levels of high parathyroid hormone (PTH), low 1-25 hydroxy vitamin D, and hypercalcemia which result in high levels of angiogenic growth factors [6]. Among patients with PHPT undergoing parathyroidectomy, Cinamon et al. found a 5% incidence of PTC and suggested that patients with PHPT should be considered at risk for PTC [7]. A similar case report from the Mayo Clinic was published in the Journal of Endocrine Practice. The authors reported a case of a 35-year-old female who underwent parathyroidectomy for primary hyperparathyroidism. Her pathology results showed a parathyroid adenoma and incidental finding of a small adjacent lymph node containing metastatic papillary thyroid carcinoma. The authors noted that careful thyroid evaluation should be considered for all patients with primary hyperparathyroidism as thyroid and parathyroid disease can coexist in the same patient [8]. There have also been reports linking secondary HPT with thyroid cancer, which adds to the evidence that hyperparathyroidism may indeed contribute to thyroid cancer risk [9-11]. Thus our patient case raises an important question as to whether patients with PHPT should undergo thorough thyroid workup prior to surgery or flexible surgical planning with thyroid nodule sampling during surgery.

In case 2, we present a patient with two consecutive benign FNA results of the same solid thyroid nodule in which the third FNAB raised concerns for malignancy. FNAB is the most valuable and cost-effective diagnostic procedure for thyroid cancer. Nevertheless, there is a wide range of diagnostic accuracy between 70 and 97%, and it is dependent on both the skill of the person performing the procedure and the pathologist interpreting the results [12]. Additionally, approximately 17 to 20% of FNAB results are classified as insufficient samples or false-negative results [3,12]. Given this limitation, repeated FNABs are usually performed for suspicious thyroid nodules. Repeated biopsies, however, may still yield unsatisfactory results in 7% of the nodules [13]. This case raises an important question as to what point in the management of patients with thyroid nodules of potential neoplastic risk, despite negative FNAB results, should we recommend surgery versus continuing close monitoring?

Our third patient presented with an atypical scalp lesion as the primary manifestation of poorly differentiated thyroid carcinoma. Cutaneous metastasis from thyroid carcinoma is rare. It usually occurs as a result of disseminated neoplastic disease; however, it may be the first indication of occult thyroid cancer as well [14]. According to Dahl et al., papillary carcinomas accounted for the majority of thyroid cancers causing skin metastases (41%), followed by follicular carcinomas with 28% and anaplastic carcinomas with 15% [15]. In contrast, Koller et al. reported that follicular carcinomas are more likely than papillary carcinomas to spread to the skin [16]. In most cases, thyroid carcinoma skin metastases occur on the scalp. Metastatic deposits can appear as flesh-colored nodules that are tender, itchy, and ulcerated [14].

Poorly differentiated thyroid carcinoma (PDTC) is a rare aggressive thyroid cancer of follicular cell origin that accounts for 4-7% of all thyroid cancers [17]. It typically presents as a neck mass with rapid growth causing compressive symptoms of nearby structures. In PDTC, skin metastases are a rare occurrence, representing 0.7-2.0% of all cutaneous malignant neoplasms. Immunohistochemical staining of skin lesions may be positive for TTF-1 and/or thyroglobulin [17].

## Conclusions

Thyroid carcinoma is the most common endocrine malignancy. This case series identifies a few unusual presentations of thyroid cancer. Larger population studies of patients with hyperparathyroidism are needed to assess whether the condition is a significant risk factor for PTC. Patients with highly suspicious thyroid nodules with negative FNAs have been found to have underlying thyroid carcinoma. Further studies are needed to determine at what point in management we should consider these patients for surgical removal despite negative FNAs. Finally, it is essential to consider the possibility of metastatic thyroid carcinoma when investigating a flesh-colored skin nodule, particularly in the scalp area.
